# Mutational spectrum and phenotypic variability of VCP-related neurological disease in the UK

**DOI:** 10.1136/jnnp-2015-310362

**Published:** 2015-06-23

**Authors:** S Figueroa-Bonaparte, J Hudson, R Barresi, T Polvikoski, T Williams, A Töpf, E Harris, D Hilton-Jones, R Petty, T A Willis, C Longman, C F Dougan, M J Parton, M G Hanna, R Quinlivan, M E Farrugia, M Guglieri, K Bushby, V Straub, H Lochmüller, T Evangelista

**Affiliations:** 1 Department of Neurology, Hospital de la Santa Creu i Sant Pau, and Universitat Autónoma de Barcelona, Barcelona, Spain; 2 The John Walton Muscular Dystrophy Research Centre and MRC Centre for Neuromuscular Diseases, Institute of Genetic Medicine, Newcastle University, Newcastle upon Tyne, UK; 3 Rare Diseases Advisory Group Service for Neuromuscular Diseases, Muscle Immunoanalysis Unit, Dental Hospital, Newcastle upon Tyne, UK; 4 Institute of Neuroscience, Newcastle University, Newcastle upon Tyne, UK; 5 Department of Neurology, Royal Victoria Infirmary, Newcastle upon Tyne, UK; 6 The John Walton Research Centre and MRC Centre for Neuromuscular Diseases, Institute of Genetic Medicine, Newcastle University, Newcastle upon Tyne, UK; 7 Department of Neurology, John Radcliffe Hospital, Oxford, UK; 8 Department of Neurology, Southern General Hospital, Glasgow, UK; 9 The Robert Jones and Agnes Hunt Orthopaedic Hospital, Oswestry, UK; 10 West of Scotland Regional Genetics Service, Southern General Hospital, Glasgow, UK; 11 The Walton Centre for Neurology and Neurosurgery, Liverpool, UK; 12 UCL MRC Centre for Neuromuscular Disease, Institute of Neurology and National Hospital for Neurology and Neurosurgery, Queen- Square, London, UK; 13 MRC Centre for Neuromuscular Disease and National Hospital for Neurology and Neurosurgery, London, UK

**Keywords:** CLINICAL NEUROLOGY, DEMENTIA, NEUROMUSCULAR, MOTOR NEURON DISEASE, MOLECULAR BIOLOGY

## Introduction

Hereditary inclusion body myopathy (IBM) with Paget's disease of the bone (PDB) and frontotemporal dementia (FTD) (IBMPFD) is a rare autosomal dominant disorder due to mutations in the valosin-containing protein gene (*VCP*).^1^ Pathogenic VCP variants have also been associated with amyotrophic lateral sclerosis^2^ and other phenotypes including dilated cardiomyopathy and Parkinson's disease. We describe phenotypic and genetic findings of 42 individuals from 21 families with VCP mutations. As our service is the reference laboratory for the UK, we calculated the UK's point prevalence based on the 2011 Census as the number of cases per population.

## Results

In total, 42 individuals were identified, 23 men and 19 women from 21 kinships (see online supplementary tables S1A, B). Based on our data, the expected point prevalence of IBMPFD in the UK is 0.066/100 000 population.

Eighteen unrelated patients harbour a previously described mutation. In addition, three patients from two families harbour two novel variants (c.604G>T, p.G202W in exon 6 and c.1316C>G, p.A439G in exon 11) that are predicted to be pathogenic by in silico analysis (Alamut interpretation software V.2.4) and segregate with disease. Three previously described mutations were identified in exon 5 of the VCP gene. The mutation p.R155H (c.464G>A) was found in 11 families. The mutation p.R191Q (c.572G>A) was found in three unrelated patients, p.R155C (c.463C>T) in two families, and p.R93C (c.277C>T) in two unrelated patients (see online supplementary material genetic analysis and mutation analysis).

The mean age of disease onset was 42.05±7.94 years. Three individuals were tested as part of a family screening and were asymptomatic at the ages of 21, 23 and 31 years.

**Figure 1:**
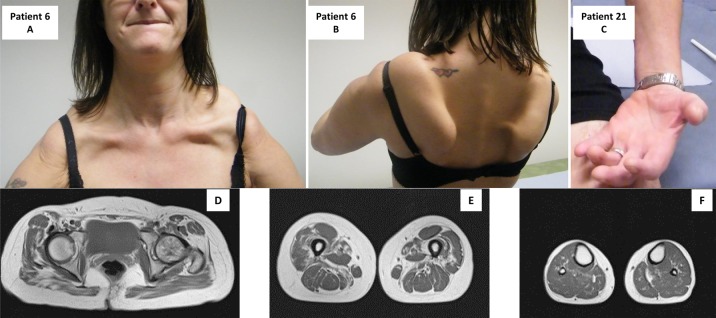
Patient 6 illustrates a pseudo-FSHD pattern. Note the scapular involvement (A) and pronounced scapular winging (B). Patient 21: note the wasting of the forearm and thenar eminence (C). Muscle MRI of pelvic girdle (D), thigh (E) and lower leg (F), showing mild fatty infiltration of Gluteus, more pronounced fatty infiltration of vastus lateralis, vastus medialis, adductor magnus and sartorius.

Muscle weakness was the first manifestation (figure 1) in 92.3% of patients, PDB was the first symptom in one case, and in two cases we were unable to obtain this information. Proximal weakness of both limb girdles was the presentation in 27% of patients; 21.6% presented with proximal upper limb weakness, and 13.5% with proximal lower limb weakness. A combination of distal and/or proximal upper and/or lower limb weakness at onset was seen in 24.2%. In two patients, falls were the first reported symptom.

Twenty-three patients remained ambulant after 15.7±8.2 years (range 5–39). The mean time to loss of ambulation was 13.37±6.6 years (range 5–22 years). Of the 10 non-ambulant patients, 6 (60%) experienced some degree of cognitive decline compared with 33.3% of the ambulant patients.

Additional clinical findings (see online supplementary table S2) were scapular winging in 20 patients (50%), markedly atrophic hands in 6 patients (15.4%), camptocormia, or bent spine, in 6 (15.4%), finger extensor weakness in 5 (12.8%), facial weakness in 3 (7.7%) and weakness of abdominal muscles in two patients (5.1%). An asymmetric pattern of muscle involvement was present in nine patients (23%). Nine patients (23%) experienced sphincter or erectile dysfunction. Two patients were diagnosed with rheumatoid arthritis and two with Parkinson's disease. Back pain (4 patients), cramps and muscle pain (12 patients) were frequently reported.

Forced vital capacity systematically assessed in 20 patients, was reduced in 2, requiring non-invasive ventilation after, respectively, 16 and 18 years of disease duration. ECG was performed in 14 patients. Three patients from the same family had moderate left ventricular dysfunction, 10, 11 and 17 years after the first symptoms. For the last patient, there was a past history of myocardial infarct (MCI). Severe progressive FTD or MCI was observed in 14 of 29 patients (48.2%); in 5 patients, data were not available. PDB was confirmed in eight patients, and five other reported bone pain. In three cases, despite normal X-rays, bone alkaline phosphatase serum levels were high.

Serum creatine kinase (CK) levels were measured in 19 of 40 patients. In 13 patients (68.4%), CK levels were mildly raised with a mean value of 379.6±142.1 U/L (N=0–150), and a range between 162 and 725 U/L. Neurophysiological data were available for 19 of 39 patients. Myopathic changes were reported in 10 of these patients, 5 presented a neurogenic pattern and 4 patients, a mixed myopathic and neurogenic pattern. Muscle biopsies, available for 17 patients, showed mild, unspecific myopathic changes except for 1, which showed a dystrophic pattern. Eleven biopsies (61%) revealed rimmed vacuoles.

## Discussion

At present, only 43 families with VCP mutations have been reported worldwide to the Leiden Open Variation Database (LVOD). Our data indicate a point prevalence of IBMPFD of 0.066/100 000 for the UK population as a whole. Although we accept that these figures need to be interpreted with caution, and cases may remain unrecognised, they suggest that IBMPFD is a very rare disease. As our department is a specialised service for muscle diseases, it is not surprising that muscle weakness was the first symptom in the majority of patients; with either a limb-girdle or combined proximal-distal distribution. Distal weakness, mostly affecting the small hand muscles, generally an extremely rare presentation of myopathy, was identified in several patients with IBMPFD.

Approximately half of our patients remained ambulant after 17.8±7.5 years. Dementia, or MCI, seems to correlate with the severity of the disease, as 60% of our patients with dementia or MCI were non-ambulant, compared with 33.3% of the patients without cognitive decline. This data on rate of progression is similar to previous reports.^3^

By comparison, the number of patients with respiratory or cardiac insufficiency was relatively low. They constitute the main cause of death in IBMPFD,^3^ and should be regularly monitored.

The presence of sphincter and erectile dysfunction, and of Parkinson's disease, further expands the phenotypic characteristics of IBMPFD. Parkinson's disease has recently been recognised as a clinical manifestation of VCP-related disease.^3^ ^4^

The presence of rimmed vacuoles, although non-specific, remains the major histological hallmark of IBMPFD.

The identification of mutations in different exons emphasises that full gene sequencing is required to exclude VCP-related disease. Owing to multisystem involvement, this disease should perhaps be called VCP-related disease to try to encompass the muscle, bone and central nervous system manifestations.

## Supplementary Material

Web supplement

Web table 1

Web table 2
