# Supplementary material for: Mutational spectrum and phenotypic variability of VCP-related neurological disease in the UK
Source: J Neurol Neurosurg Psychiatry. 2015 Jun 23;87(6):680–1. doi: 10.1136/jnnp-2015-310362 (PMC4893144; doi:10.1136/jnnp-2015-310362)
Supplement: Web supplement [file jnnp-2015-310362-s1.pdf]

Genetic Analysis

DNA samples were analyzed by bi-directional fluorescent sequencing of all 17 exons of the VCP gene. Primer sequences were designed using the Primer-3 Plus program and are available upon request. PCR reaction mix consisted of 50ng genomic DNA, 0.1 µmol of each primer, and Immolase (Bioline) in a reaction mix of 20µl. Amplification of exon 1 contained an additional 15% DMSO. Denaturation was performed at 95°C for 4 min and then followed by 32 cycles of 94°C for 1min, 60°C for 1min, and 72°C for 1min, with a final extension step of 72°C for 10 min.

PCR products were sequenced with the BigDye Terminator 3.1 Cycle Sequencing Kit (Applied Biosystems). Sequencing reactions were size-separated on an Applied Biosystems 3500 xL Genetic Analyzer. Sequence data was analyzed with Mutation Surveyor version 4.0.8 software (Soft Genetics). Nomenclature was based on sequence accession number NM_007126.3 where the A of the initiation codon ATG is nucleotide 1.

Mutation analysis

In addition, three unrelated patients referred for VCP testing were found to have variants of unknown clinical significance: c.1360-14C>G, c.2214A>G and c.2316-46C>T. None of these three patients had a clinical phenotype strongly suggestive of IBMPFD. The c.2214A>G synonymous substitution is not predicted by bioinformatic analysis to be pathogenic. The intronic variants c.1360-14C>G and c.2316-46C>T are predicted by bioinformatic analysis to have a potential impact on splicing and we are aiming to undertake RNA studies to investigate these variants further once samples become available.
