# Supplementary material for: Mutational spectrum and phenotypic variability of VCP-related neurological disease in the UK
Source: J Neurol Neurosurg Psychiatry. 2015 Jun 23;87(6):680–1. doi: 10.1136/jnnp-2015-310362 (PMC4893144; doi:10.1136/jnnp-2015-310362)
Supplement: Web table 1 [file jnnp-2015-310362-s2.pdf]

Table 1.a – Demographic, clinical and molecular data

Family no. or sporadic case; hereditary pattern	Case no.	Gender	Age/Age at onset (y)	WCB (y after onset)	Mode of onset (muscle weakness)	Follow-up (muscle weakness)	Mutation
I; AD	1	M	21 / **(*)**	**(*)**	**(*)**	**(*)**	p.R155C
	2	F	45/35	Yes (6 y)	Proximal UL	Proximal: UL/LL; Distal: UL>LL	p.R155C
	3	F	ψ 55/40	No	Proximal UL	Proximal: UL/LL	p.R155C
	4	M	ψ 56/39	Yes(7 y)	Proximal UL	Proximal: UL/LL	p.R155C
	5	M	ψ 63/40	Yes(22 y)	Proximal LL	Proximal: UL/LL	p.R155C
II; AD	6	F	41/33	No	Proximal UL / LL	Proximal UL/LL	p.R155H
	7	M	ψ 59/53	No	Distal LL	Distal: UL/LL	p.R155H
	8	F	31/**(*)**	**(*)**	**(*)**	**(*)**	p.R155H
	9	F	59/30	No	Proximal LL	Proximal: UL/LL	p.R155H
III; AD	10	M	56/40	No	Proximal UL / LL	Proximal UL/LL	p.R155H
	11	M	52/34	No	Proximal UL / LL	Proximal UL/LL; Distal LL>UL	p.R155H
	12	F	73/55	Yes(10 y)	Proximal UL / LL	Proximal UL/LL	p.R155H
	13	M	54/28	Yes(20 y)	Proximal UL / LL	Proximal: UL/LL; Distal: LL>UL	p.R155H
	14	F	57/35	No	Proximal UL / LL	Proximal: UL/LL; Distal: LL>UL	p.R155H
	15	F	52/42	No	Proximal UL	Proximal: UL/LL	p.R155H
	16	F	56/30	No	Proximal UL	Proximal: UL/LL; Distal: UL/LL	p.R155H
	17	F	53/33	No	Proximal UL / LL	Proximal: UL/LL; Distal: UL>LL	p.R155H
	18	M	38/31	Yes(5 y)	Proximal LL	Proximal: UL/LL; Distal: UL>LL	p.R155H
Case; sporadic	19	M	54/32	Yes(19 y)	Distal UL / LL	Proximal: UL/LL; Distal: UL/LL	p.R155H
Case; sporadic	20	F	66/52	No	Proximal UL	Proximal: UL>LL; Distal: UL/LL	p.R155H
Case; sporadic	21	M	53/45	No	Distal UL	Proximal: UL/LL; Distal: UL>LL	p.R191Q
Case; sporadic	22	M	56/42	No	Distal LL	Proximal: UL/LL; Distal: LL>UL	p.R93C
Case; sporadic	23	M	62/54	No	Distal LL	Distal: LL>UL, Proximal: UL>LL	p.R155C
IV; AD	24	M	ψ/(-)	(-)	(-)	(-)	p.R155H
	25	M	23/**(*)**	**(*)**	**(*)**	**(*)**	p.R155H
Case; sporadic	26	M	57/48	No	Proximal UL / LL	Proximal: LL>UL	p.R155H
Case; sporadic	27	M	46/35	No	Proximal UL / LL	Proximal: UL/LL + distal LL	p.R155H
Case; sporadic	28	F	58/49	No	Proximal UL	Proximal + distal: UL; Proximal: LL	p.R155H
Case; sporadic	29	F	Ψ56/36	No	(-)	(-)	p.R155H
Case; sporadic	30	F	50/42	No	Falls;	Proximal UL>LL	p.R155H
Case; sporadic	31	F	55/46	No	Proximal UL / LL	Proximal UL<LL	p.R155H
Case; sporadic	32	M	63/54	No	Proximal LL	Proximal UL/LL	p.R191Q
Case; sporadic	33	F	ψ67 /48	No	Distal LL>UL	Proximal LL>UL ; distal UL/LL	p.R93C
Case; sporadic	34	M	62/48	(-)	Proximal UL / LL	Proximal UL/LL; Distal UL	p.R191Q
Case, AD - probable	35	F	Ψ78/50	Yes(-)	Proximal UL; Distal LL	Proximal +distal: UL/LL	p.G202W
V; AD – probable	36	M	79/40	No	Proximal UL	Proximal: UL> LL Distal: LL> UL	p.A439G
	37	M	ψ66/41	No	Distal UL	Proximal: UL/LL	p.A439G
VI; AD	38	M	56 / 42	No	Proximal LL	Proximal: LL/UL	p.R155H
	39	F	63 / 58	No	Falls	Proximal: LL>UL; Distal: LL	p.R155H
	40	F	57 / 38	No	Distal UL	Distal> proximal: UL and LL	p.R155H
	41	M	Ψ58/40	Yes (18)	Proximal LL	Generalized UL>LL, Proximal>distal	p.R155H
	42	M	Ψ61/(60)	Yes (-)	(-)	(-)	p.R155H

Legend: F, female; M, male; y: years; **(*)**, asymptomatic; ψ, deceased; WCB, Wheelchair Bond; UL, upper limbs; LL, lower limbs; (-), missing data; AD, autosomal dominant

Table 1.b– Demographic, clinical and molecular data

Case no	Last FVC	Echocardiogram	Cognitive impairment	Sphincter involvement	CK U/L (N<150)	PDB /High ALP	Mutation
2	Normal	(-)	Yes	No	Normal	(-)	p.R155C
3	(-)	(-)	No	No	Normal	(-)	p.R155C
4	(-)	(-)	Yes	No	(-)	(-)	p.R155C
5	(-)	(-)	Yes	No	350	(-)	p.R155C
6	94%	(-)	No	No	(-)	Yes/Yes	p.R155H
7	(-)	(-)	(-)	No	(-)	Yes/Yes	p.R155H
9	(-)	(-)	No	No	Normal	(-)	p.R155H
10	101%	Normal	(-)	Yes	500	(-)	p.R155H
11	76%	Reduced left ventricle function	(-)	Yes	500	No/Yes	p.R155H
12	109%	Moderate left ventricle systolic dysfunction	Yes	Yes	280	No/Yes	p.R155H
13	111%	Moderate left ventricle systolic dysfunction	No	Yes	238	(-)	p.R155H
14	50%	(-)	No	Yes	(-)	(-)	p.R155H
15	119%	(-)	No	Yes	(-)	(-)	p.R155H
16	132%	(-)	No	No	(-)	Yes/Yes	p.R155H
17	Normal	(-)	No	Yes	(-)	(-)	p.R155H
18	80%	(-)	No	No	725	(-)	p.R155H
19	Normal	(-)	Yes	No	299	(-)	p.R155H
20	Normal	(-)	No	No	287	(-)	p.R155H
21	111%	(-)	No	No	(-)	(-)	p.R191Q
22	107%	(-)	Yes	No	400	No/Yes	p.R93C
23	75%	Normal	Yes	No	Normal	Yes/Yes	p.R155C
24	(-) Respiratory involvement	(-)	(-)	No	(-)	(-)	p.R155H
26	100%	(-)	No	No	(-)	Yes/Yes	p.R155H
27	100%	Abnormal	No	No	500	(-)	p.R155H
28	(-)	(-)	No	No	Normal	(-)	p.R155H
29	(-)	(-)	Yes	No	(-)	Yes/Yes	p.R155H
30	(-) Respiratory involvement	Normal	(-)	No	Normal	(-)	p.R155H
31	100%	(-)	Yes	No	Normal	No/No	p.R155H
32	85%	Normal	Yes	No	(-)	Yes/Yes	p.R191Q
33	86%	Normal	Yes	Yes	Normal	No/No	p.R93C
34	100%	Normal	Yes	No	(-)	No/No	p.R191Q
35	100%	Normal	Yes	No	Normal	(-)	p.G202W
36	100%	Normal	(-)	No	391	No/No	p.A439G
37	(-)	(-)	Yes	Yes	162	No/No	p.A439G
38	(-)	Normal	No	No	(-)	(-)	p.R155H
39	97%	(-)	(-)	(-)	303	(-)	p.R155H
40	78%	Normal	No	No	Normal	(-)	p.R155H
41	Ventilated	Normal	No	No	500	(-)	p.R155H
42	(-)	(-)	(-)	(-)	(-)	(-)	p.R155H

Legend: F, female; M, male; y, years; PDB, Paget disease of the bone; ALP, Alkaline Phosphatase; FVC, Forced Vital Capacity (% of predicted); (-), missing data;
