# Supplementary material for: Mutational spectrum and phenotypic variability of VCP-related neurological disease in the UK
Source: J Neurol Neurosurg Psychiatry. 2015 Jun 23;87(6):680–1. doi: 10.1136/jnnp-2015-310362 (PMC4893144; doi:10.1136/jnnp-2015-310362)
Supplement: Web table 2 [file jnnp-2015-310362-s3.pdf]

Table 2 – Additional clinical findings

Additional clinical findings	(n)	%
Scapular winging	20	50%
Muscle pain and/or cramps	12	30.7%
Asymmetrical involvement	9	23%
Sphincter dysfunction	9	23%
Camptocormia or bent spine	6	15.4%
Atrophic hands	6	15.4%
Finger extensors weakness	5	12.8%
Autonomic, sensory and motor neuropathy	3	7.8%
Facial weakness	3	7.7%
Abdominal wall weakness	2	5.1%
Deafness	2	5.1%
Rheumatoid arthritis	2	5.1%
Ptosis + external ophtalmoparesis	1	2.6%
Bull’s eye maculopathy	1	2.6%
Gynecomastia	1	2.6%
Sleep apnoea	1	2.6%
Rigid spine	1	2.6%
Hand tremor	1	2.6%
Parkinson’s Disease	1	2.6%

Legend: Additional clinical findings reported in our cohort. (n) Number of affected. % = (n)/39
